# Sputtered Encapsulation as Wafer Level Packaging for Isolatable MEMS Devices: A Technique Demonstrated on a Capacitive Accelerometer

**DOI:** 10.3390/s8117438

**Published:** 2008-11-19

**Authors:** Azrul Azlan Hamzah, Jumril Yunas, Burhanuddin Yeop Majlis, Ibrahim Ahmad

**Affiliations:** 1 Institute of Microengineering and Nanoelectronics (IMEN), Universiti Kebangsaan Malaysia, 43600 Bangi, Selangor, Malaysia; E-mail: azlanhamzah@ukm.my; burhan@vlsi.eng.ukm.my; 2 Department of Electrical, Electronics, and Systems Engineering, Universiti Kebangsaan Malaysia, 43600 Bangi, Selangor, Malaysia; E-mail: ibrahim@vlsi.eng.ukm.my

**Keywords:** Silicon encapsulation, sputtering, wafer level packaging, capacitive accelerometer, liquid crystal polymer

## Abstract

This paper discusses sputtered silicon encapsulation as a wafer level packaging approach for isolatable MEMS devices. Devices such as accelerometers, RF switches, inductors, and filters that do not require interaction with the surroundings to function, could thus be fully encapsulated at the wafer level after fabrication. A MEMSTech 50g capacitive accelerometer was used to demonstrate a sputtered encapsulation technique. Encapsulation with a very uniform surface profile was achieved using spin-on glass (SOG) as a sacrificial layer, SU-8 as base layer, RF sputtered silicon as main structural layer, eutectic gold-silicon as seal layer, and liquid crystal polymer (LCP) as outer encapsulant layer. SEM inspection and capacitance test indicated that the movable elements were released after encapsulation. Nanoindentation test confirmed that the encapsulated device is sufficiently robust to withstand a transfer molding process. Thus, an encapsulation technique that is robust, CMOS compatible, and economical has been successfully developed for packaging isolatable MEMS devices at the wafer level.

## 1. Introduction

Nowadays device packaging remains a major challenge in the micro electromechanical systems (MEMS) industry. The vast variation in shape and function of MEMS devices make it virtually impossible to create universal packages like those used in integrated circuits, while maintaining the integrity and functionality of the devices. Many application specific MEMS devices such as accelerometers, pressure sensors, and microgyroscopes require custom packaging to function. Microsensors often require contact with the surroundings to measure air or fluid pressure, gas content, or flowing liquids, making device packaging even more challenging and expensive. On top of being geometrically complex to package, moving parts of MEMS devices are highly sensitive to damage and contamination during fabrication and packaging processes. Damage could be caused by chemical contamination, physical touch, or contamination by micro dirt [1]. The custom packaging and special handling needed drive packaging cost high. Moreover, stringent device performance requirements have driven the need for a packaging method that is robust, manufacturable using CMOS compatible processes, and easily fabricated and tested. In an effort to lower packaging cost, improve yield, and comply with these requirements, many MEMS manufacturers are starting to explore wafer level packaging.

Wafer level packaging is an approach at which the dies are individually encapsulated on wafer level before dicing. Using this approach, MEMS devices generally require two levels of packaging [2]:

i) Wafer level packaging: at this level, encapsulation is applied simultaneously on all dies to provide protection during handling, dicing and testing. This type of packaging is usually hermetic.
ii) Conventional packaging: after encapsulation is applied, the wafer is diced. Then, each die is picked, placed, and bonded on a leadframe, wire bonded, and plastic molded. This type of packaging is usually non-hermetic.

A typical MEMS device consists of sensor or actuator elements fabricated on a silicon substrate. MEMS devices such as RF switches, inductors, filters, and accelerometers do not require interaction with the surroundings to function. Hence, a complete isolation of the sensor or actuator elements would increase device performance as well as its lifetime. A conventional wafer level packaging technique is cap bonding. The bonded cap, however, is commonly thick and occupies a lot of real estate as the cap has to be bonded over and around the movable elements of the device [1]. For this reason, deposited encapsulation is becoming more favorable for packaging these isolatable MEMS devices. In this process, movable parts of a MEMS device are covered by deposited metal or silicon encapsulation, leaving a gap between the top surface of the movable parts and the bottom surface of the encapsulation. As a result, the encapsulation allows movable elements to move freely while protecting them.

Various techniques have been developed to encapsulate isolatable MEMS devices. Partridge *et al.* have pioneered the use of epitaxially deposited polysilicon as an encapsulation structure for piezoresistive accelerometers [3]. In extension to the aforementioned work, Rusu *et al.* studied plasma-enhanced chemical vapor deposition polysilicon germanium as an alternate material for device encapsulation [4]. Meanwhile, Lebouitz *et al.* used permeable polysilicon to fabricate a vacuum shell over movable elements of a MEMS resonator [5]. Alternate methods involve using chemical vapor deposition sealed micromachined cavity and SiN microshell to protect microstructures [6-7]. However, all the aforementioned techniques involve high process temperatures that are detrimental to prefabricated CMOS circuitry, which is often damaged at temperature higher than 450°C. As a solution, a low temperature process was introduced using electroless nickel cavity as the encapsulation structure [8]. The fabrication process, however, requires an electroplating step, which is less compatible with CMOS and other pre-fabricated microstructures. In light of these problems, this work attempts to solve the wafer level packaging issues faced by isolatable devices through a simple and economical CMOS compatible microfabrication processes. A novel wafer level encapsulation is introduced, which uses sputtered silicon as main structural layer, eutectic gold-silicon alloy as seal layer, and high strength liquid crystal polymer (LCP) thermoplastic as outer encapsulant layer. A robust encapsulation which could withstand subsequent high pressure plastic packaging is produced only by using and carefully sequencing several CMOS compatible materials and low temperature processes, without the need for expensive packaging procedures and equipments.

## 2. Methodology

In order to assess the feasibility of the proposed packaging technique, sputtered silicon encapsulation was built on an unpackaged MEMSTech 50g non-crossing differential capacitive accelerometer (Sensfab Pte. Ltd., Singapore). This device is selected due to its size suitability. The device has an adequately large movable elements area for the encapsulation to be built upon. For this reason, viability of the technique on this device would imply its feasibility on other MEMS devices with smaller movable elements area, since all the CMOS compatible processes used could be scaled down. Moreover, the device structure is planar, yet fairly complicated, which made it practical to gauge the potential of this packaging technique.

**Figure 1.**
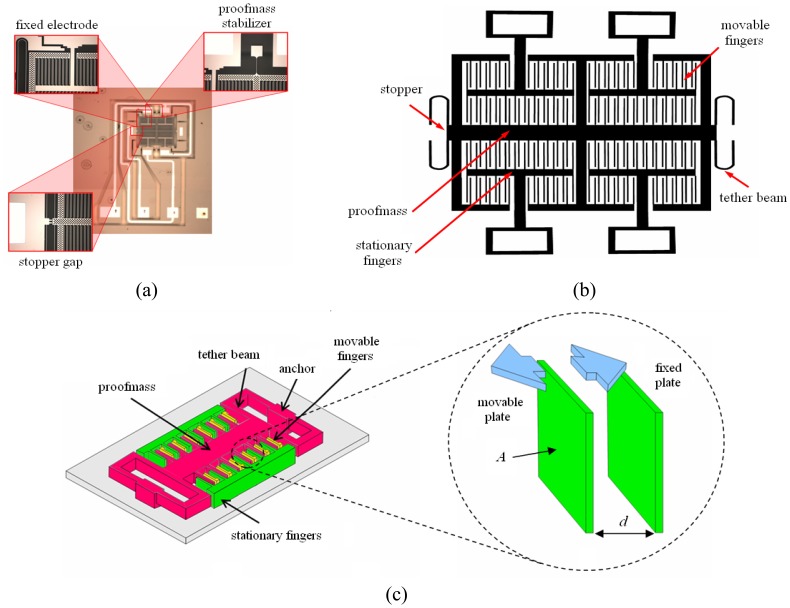
(a) Optical microscope picture showing stopper gap, proofmass, and electrode, (b) schematic diagram showing overall layout and components, and (c) 3-D functional model of MEMSTech 50g accelerometer.

The accelerometer device has four identical quadrants of accelerometer fingers, with 84 pairs of beams per side, as shown in Figures 1(a) and 1(b). The device has a set of stationary fingers at each quadrant and a proof mass holding all the movable fingers [Figure 1(b)]. It has sensor die area of 2,350 × 3,020 μm^2^, which includes bond pad area of 652.3 μm × 2,331.7 μm, and movable elements area of about 500 μm × 700 μm. The encapsulation structure has to cover all movable elements and have a decent base to be built upon. Considering the position of traces and isolation trenches, it was determined that the most feasible size for the encapsulation structure is 1,455 μm × 1,455 μm. Encapsulation of this size would envelop over all movable elements and critical traces while having appreciable base area to stick to. The inner cavity would have a dimension of 1,250 μm × 1,250 μm, and height of approximately 5 μm. The difference between outer and inner dimension, which is 205 μm, would provide a base of width 102.5 μm around the perimeter of the encapsulation structure.

Capacitive accelerometer detects the variance of capacitance resulting from movement of the parallel plates due to impact, as depicted in Figure 1(c). Capacitance (*C*) of a pair of parallel plates is given by:

(1) $$C=\frac{\varepsilon_{s}A}{d}$$

where *A* is the overlapping area of the plates, *d* is the spacing between the plates, and *ε_s_* is the static permittivity of the material between the plates. For the 50g accelerometer used, the static permittivity is close to vacuum permittivity (*ε_0_*) since the gap between the plates is occupied by air. The accelerometer has an absolute capacitance value of approximately 1 pF.

Several tests are conducted on the encapsulation structure after fabrication. The encapsulated device was visually inspected for defects and microstructure release using a scanning electron microscope (SEM) and optical microscopy. Then, a profile scan is performed across the encapsulation using a *Tencor* surface profiler to check for surface uniformity and existence of microcracks on the structure. After outer encapsulant application, strength of the dome shaped outer encapsulant layer was determined using a Micro Materials' (Wrexham, U.K.) *NanoTest* indenter system, which measures hardness and Young's modulus of the outer encapsulant. Seven identical cured encapsulant samples were used in the strength measurement test, with one indent per sample. The final test performed on the encapsulated device was a capacitance test, which was measured using an Agilent 4284A Precision LCR Meter. As shown by Equation (1), the capacitance value depends on the static permittivity, which varies depending on the material occupying the gap between the parallel capacitor plates. Therefore, complete release of sacrificial material can be verified by comparing capacitance value before and after encapsulation fabrication. 11 samples are tested before and after encapsulation and capacitance values are compared

## 3. Encapsulation Fabrication Process

**Figure 2.**
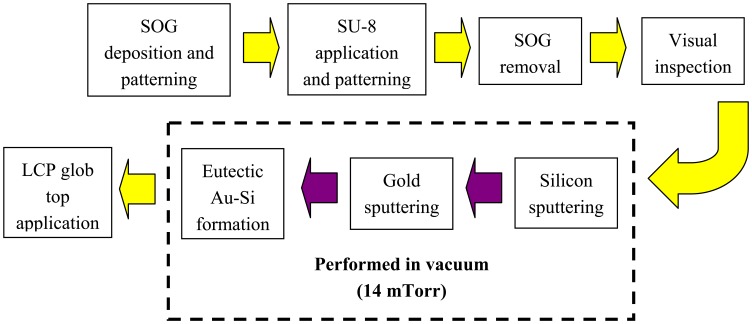
Process sequence for sputtered silicon encapsulation fabrication.

**Figure 3.**
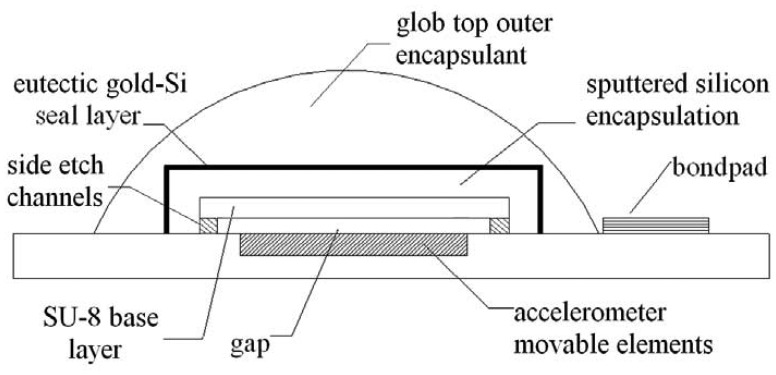
Schematic diagram of encapsulated MEMSTech 50g accelerometer.

Encapsulation fabrication involves several deposition and etching steps. The process is initialized by sacrificial spin-on glass (SOG) deposition and patterning, and followed by SU-8 base layer formation, sacrificial SOG removal, visual inspection of release etch, silicon main structural layer formation, gold layer deposition, eutectic gold-silicon seal layer formation, and LCP glob top outer encapsulant application, all in the stated order. These process steps are summarized in Figure 2. Schematic showing different parts of completed wafer level packaging for MEMSTech 50g accelerometer is depicted in Figure 3.

### 3.1. Sacrificial Layer Formation

Accuglass 314 SOG from Honeywell Inc. (Sunnyvale, USA) was used as sacrificial layer. This material was chosen due to its ease of application, ease of etching back in hydrofluoric acid (HF), relatively cheap price, good planarization properties, and its similar characteristics to conventionally deposited silicon oxide [9]. Seven consecutive layers of SOG were applied on the accelerometer's movable elements. Each layer was spun at 2,000 rpm for 30 seconds and baked at 200°C for 4 minutes. The samples were then etched back in 1% HF solution (1:100 HF-H_2_O) under mild agitation for approximately 24 minutes. Square SOG pattern with narrow rectangles around its perimeter for side etch channels formation was fabricated.

### 3.2. Encapsulation Base Formation

Immediately after SOG sacrificial layer formation, a single SU-8 (type 2007) layer was spun, patterned, and developed on top of the SOG sacrificial layer. SU-8 is used as the base layer for the encapsulation. The SU-8 layer was spun at 2,000 rpm and consecutively baked at 95°C for 2 minutes. A square mask was aligned over the SOG layer using a UV mask aligner. The mask was aligned such that part of the edges of the SOG perimeter rectangles protruded from underneath the SU-8 layer. The protruding SOG would initiate sacrificial layer removal in the subsequent process step. After post exposure bake (PEB), the SU-8 layer was annealed at 200°C for 3 hours using direct hotplate baking. The annealing would further densify and harden the polymer so that it is adequately strong to endure subsequent furnace annealing step, and function as structural material of the encapsulation.

### 3.3. Release Etch and Visual Inspection

The most critical step in determining the functionality of the device after encapsulation is the release etch. The etch release step was performed using 5% HF solution (1:20 HF-H_2_O). This solution was found effective in removing sacrificial SOG, while being less damaging to both the SU-8 base layer and silicon structure of the accelerometer [10].

**Figure 4.**
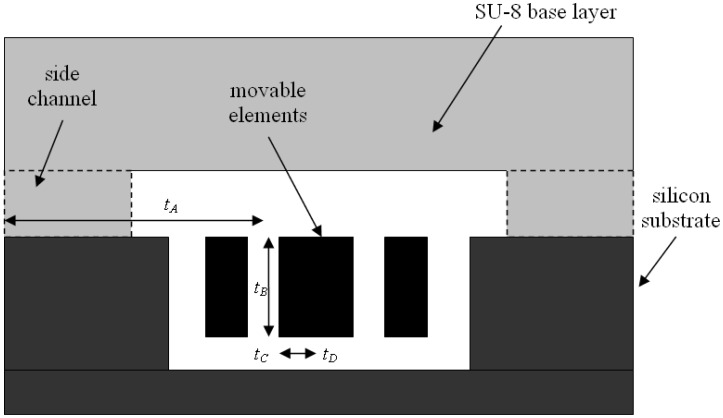
Schematic diagram of a base encapsulated MEMSTech 50g accelerometer showing total etch time needed to completely release the accelerometer movable elements.

Optimal etch time needed to be determined for complete removal of sacrificial oxide. For the accelerometer device selected, total etch time is the summation of etch times at all the subsections. Maximum etch distance needs to be taken into account. Schematic of SU-8 encapsulated 50G accelerometer with side etch channels is depicted in Figure 4 below. It could be seen that total etch time could be formulated as follows:

(2) $$T_{\text{total}}=t_{A}+t_{B}+t_{C}+t_{D}$$

Note that *t_C_* is T-junction region. Etch rate in this section is 2/3 of the etch rate in straight sections [10]. For the 50g accelerometer device, the etch time and total etch time to completely release the structure is tabulated in Table 1 below. Upon completion of release etch step, moisture was removed from within encapsulation base structure via direct hotplate baking. The device was gradually heated to 90°C and maintained at peak temperature for 20 minutes, before being slowly cooled to room temperature. Next, owing to transparent property of cured SU-8, visual inspection of complete SOG removal was performed using optical microscope.

### 3.4. Encapsulation Main Structure Formation

Sputtered silicon was used as the main structure for the encapsulation. A silicon mask with square openings was used to pattern the sputtered silicon so as only the area underneath the openings were sputtered. The samples were aligned such that the SU-8 square base structure was at the very center of the mask square opening. The samples were then placed in *NanoFilm* (NTI Pte. Ltd., Singapore) sputtering chamber and RF silicon sputtering was performed at 400W forward power with 20 sccm argon flow, at approximately 80°C. The sputtering process was performed for a total of 23 hours and 50 minutes, at pressure level of approximately 14 mTorr, yielding a silicon layer of approximately 25 μm in height. Despite lower deposition rate, this pressure level was selected because of its higher film adhesion due to lower sputtering pressure [11].

### 3.5. Seal Layer Formation and Annealing of Silicon Layer

Immediately after silicon sputtering was completed, the samples were transferred into a Baltec DC sputtering chamber for gold sputter deposition. The sputtering process was performed continuously for approximately one hour at a pressure level of approximately 14 mTorr and 40 mA current. Then, the samples were placed in furnace and annealed at 363°C for 3 hours. The annealing served two purposes: (1) to grow a eutectic gold-silicon layer between sputtered gold and sputtered silicon layer, and (2) to anneal the amorphous sputtered silicon into a more densified layer.

The gold-silicon eutectic layer serves as the seal layer for the encapsulation. Gold-silicon eutectic layers have been widely used as hermetic bond layers in MEMS [12-14]. Eutectic layer formation is achieved by annealing both gold and silicon atoms at eutectic temperature. At a temperature of 363°C, gold atoms diffuse into the silicon layer creating metastable amorphous gold silicide. The epitaxial growth of this diffusion layer is immediately followed by hermetic eutectic gold-silicon layer formation [15]. At the eutectic temperature, silicon and gold atoms experience localized melting and locally form thin eutectic layer [15]. The eutectic layer is created between the sputtered gold and sputtered silicon layers, in the order of a few hundred nanometers thick, as the reaction is limited by the correct percentage of both silicon and gold atoms at the interface of gold and silicon layers. This thin layer is very densely uniform in terms of atomic arrangement, thus results the hermeticity of the layer [16].

### 3.6. Application of Outer Encapsulant

Outer encapsulant needs to be added on top of the encapsulation structure in order to strengthen the encapsulation structure for the subsequent transfer molding process. A thin glob of RTP 3485-1 LCP carbon fiber was melt-dispensed on encapsulated MEMSTech 50g accelerometer using a dispenser heated to approximately 300°C to melt the thermoplastic, yielding an encapsulated device with a uniform glob top after curing. During hardening, slow cooling was used to avoid stress build-up. This thermoplastic was selected as encapsulant material because of its sufficiently high Young's modulus value and its matching coefficient of thermal expansion (CTE) with the outermost gold layer. Gold, which possesses CTE value of 14 ppm/°C, could be excellently matched by the CTE value of LCP carbon fiber, which is on the range of 12-18 ppm/°C [17]. CTE matching guarantees low thermal stress build-up between gold and thermoplastic interfaces, thus reducing the possibility of post-process intrinsic stress build-up and delamination. Only a thin layer of glob, not to exceed 210 μm thick, is dispensed on the encapsulation structure to maintain the thin profile of the packaged device. For this thickness, cured LCP with Young's modulus value greater than 26.5 GPa is needed to withstand pressure up to 100 atm during subsequent plastic package transfer molding process [18].

## 4. Results and Discussion

### 4.1. Visual Inspection of the Fabricated Encapsulation

**Figure 5.**
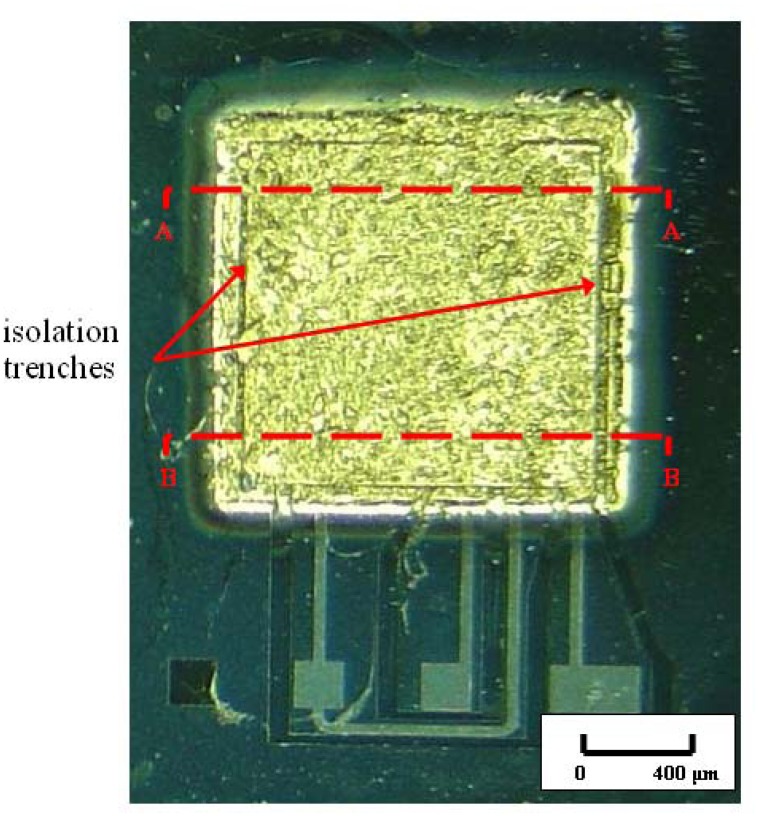
Optical microscope picture showing close up of an encapsulated MEMSTech 50g accelerometer. Imprints of isolation trenches are visible from the top of the device.

Optical microscope picture of encapsulated for MEMSTech 50g accelerometer is shown in Figure 5. In this top view photograph, golden squares could be prominently seen, since gold is the topmost layer of the encapsulation. The topmost gold layer serves as encapsulation protection layer against oxidation and other chemical attack, owing to inert properties of gold. Right underneath this layer is the eutectic gold-silicon layer, which serves as the seal layer for the structure. The subsequent layer is annealed silicon, which serves as the main structural layer of the encapsulation. The innermost layer is SU-8 layer, which serves as the base for encapsulation structure.

The encapsulation structure has a dimension of 1,455 μm by 1,455 μm. Thus, the total real estate area taken by the encapsulation is very minimal, merely 2.12 mm^2^. Upon closer inspection on an individual completed device, it could be seen that the encapsulation has been squarely fabricated on top of the movable elements of the device (Figure 5). Imprints of the underlying isolation trenches are also visible. Note that the encapsulation fabrication process has also maintained the integrity of all the traces and isolation trenches. None of the prefabricated traces or trenches is damaged during the encapsulation process.

**Figure 6.**
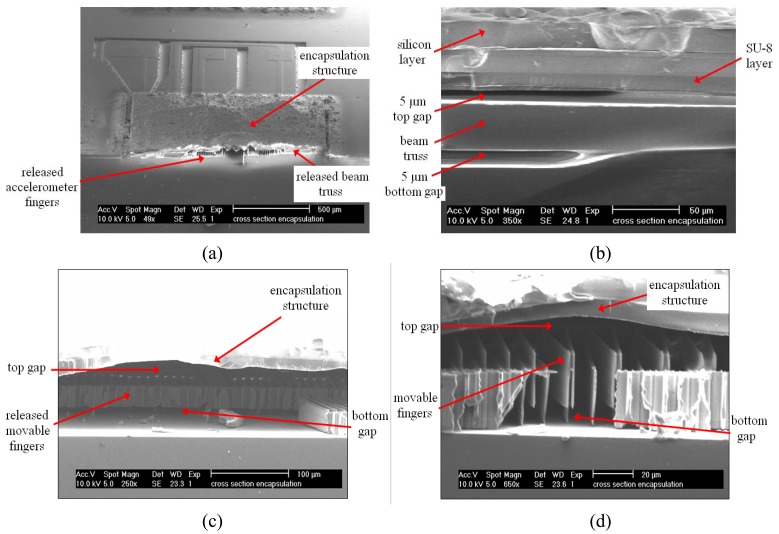
SEM micrograph showing (a) 60° view of an encapsulated MEMSTech 50g accelerometer broken through section A-A of Figure 5, (b) released beam truss, (c) released movable fingers, and (d) close-up view of released stationary and movable fingers.

Figure 6 focuses on accelerometer movable elements. Figure 6(a) captures rows of fingers and beams underneath the encapsulation structure. Figure 6(b) further focuses on a released beam truss from 90° viewing angle. The layers that constitute encapsulation could be clearly seen. Note that there are gaps of approximately 5 μm underneath and above the beam truss. Figure 6(c) shows a row of movable fingers underneath an encapsulation structure. All of the sacrificial SOG has been removed from the movable area. Similarly, gaps of approximately 5 μm exist atop and underneath the movable fingers.

A common and critical problem involved when using wet release etching is sticking. In MEMS, some of the movable elements would stick to one another after wet etch due to static force. Process wise, this problem is commonplace as long as wet etching is involved. Accelerometer fingers sticking to one another are shown in Figure 6(d) below. This phenomenon could be significantly remedied by controlled evaporation of moisture after wet etching process. Alternatively, an efficient method to avoid stiction is using vapor phase etching or creating dimples on movable elements where stiction is most likely to occur [19]. Controlled moisture evaporation was employed in the encapsulation technique introduced in this work to overcome stiction problem. The samples are placed in low humidity oven and baked at 60°C for 5 minutes with 2°C/min increment from room temperature during heating. This step is observed to eliminate stiction problem in 80% of the samples tested.

### 4.2. Scan Profile of Encapsulation Structure

**Figure 7.**
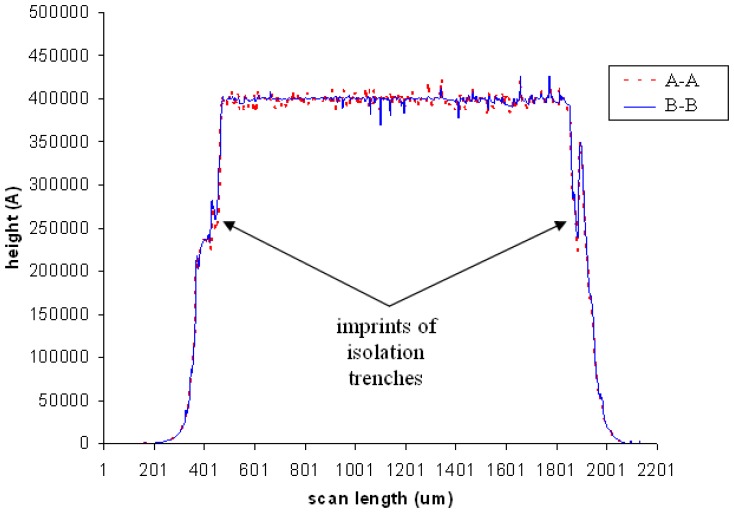
*Tencor* surface profiler scan across sections A-A and B-B of Figure 5.

A profile scan was performed across section A-A and repeated at section B-B to investigate encapsulation surface contour. A force of 5 × 10^-5^ N was applied across both surfaces. As could be seen in Figure 7, surface profiles across both sections are very homogeneous. It is thus verified that the encapsulation is very flat across its surface, with average thickness of 40 μm. The flat surface indicates the quality of fabricated base layer and sputter deposited silicon and gold layers. The deposited layers are uniformly dense across the entire structure. No substantially deficient cracks or voids are present on the surface and side walls of the encapsulation. At the onset and drop off of both side walls, steep dips are observed. These are the imprints of isolation trenches observed in Figure 5. As could be seen from the scan profile, the isolation trenches do not affect the integrity of the encapsulation structure. The total thickness of the encapsulation is the sum of thickness of the various layers constituting it. Thickness of each layer is summarized in Table 2.

### 4.3. Outer Encapsulant Strength Test

**Figure 8.**
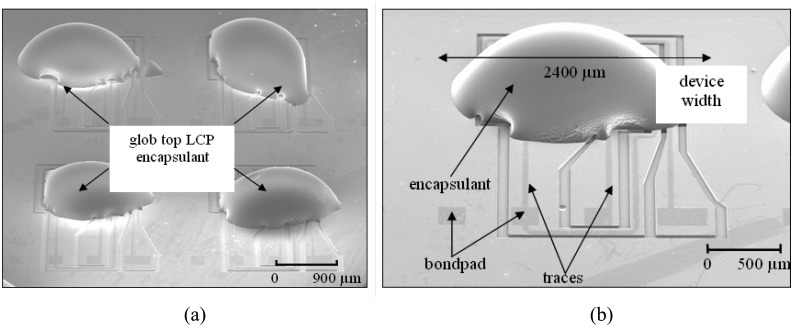
SEM micrograph showing (a) an array of LCP encapsulated MEMSTech 50g accelerometer and (b) a single 50g accelerometer encapsulated with LCP. Note that the traces and bondpads are exposed, while sputter encapsulated accelerometer fingers are enveloped underneath the outer encapsulant.

The glob top encapsulated accelerometer device is shown in Figure 8. It could be seen that the cured glob top encapsulant evenly envelop the sputtered encapsulation. However, the slight variation in shape of the glob top encapsulants is due to the manual dispensing process. Automated dispensing would indeed be used during production stage to ensure glob top uniformity. This step completes wafer level packaging of the accelerometer device.

Nanoindentation test yields indentation load-depth correlation, plastic depth, hardness, and reduced modulus of the outer encapsulant, as tabulated in Table 3. Maximum indentation depth is the distance the tester tip penetrates at the corresponding maximum load value. Plastic depth is the software estimated depth where plastic deformation starts to take place. Hardness is defined as resistance of the material to permanent deformation. Finally, reduced modulus is obtained based on elastic stress and elastic strain of the material tested.

Reduced modulus is related to Young's modulus of the material by the following correlation:

(3) $$\frac{1}{E_{r}}=\frac{1-{v_{s}}^{2}}{E_{s}}+\frac{1-{v_{i}}^{2}}{E_{i}}$$

where *E_s_* and *ν_s_* are Young's modulus and Poisson ratio of the test sample, and *E_i_* and *ν_i_* are Young's modulus and Poisson ratio of the indenter, which in our case are 1,141 GPa and 0.07 respectively [20]. The reduced modulus values tabulated in Table 3 are checked against 3-sigma control limit. It is observed that all the data points falls within upper (UCL) and lower (LCL) control limits. Therefore, the sample average could be plausibly used as actual reduced modulus value of the outer encapsulant material. Based on Table 3, average reduced modulus value is 31.22 GPa. Taking this value, assuming *ν_s_* value of 0.4 for a typical high strength thermoplastic, and plugging the values into Equation (3) yields actual Young's modulus value of 26.96 GPa for the encapsulant material. This value exceeds the minimum Young's modulus value required to withstand 100 atm pressure. Therefore, the glob top encapsulated accelerometer could safely undergo plastic package transfer molding process.

### 4.4. Capacitance Test

**Figure 9.**
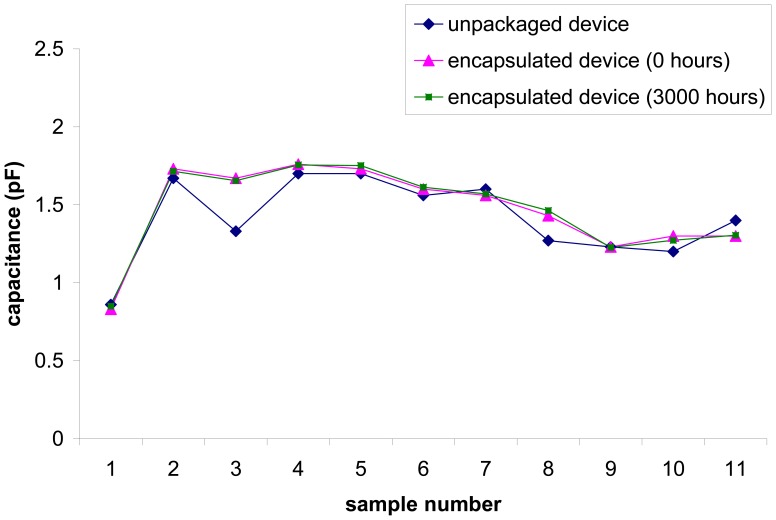
Plot showing comparison of capacitance value between unpackaged and encapsulated MEMSTech 50g accelerometer, both at 0 and 3,000 hours.

As stated in Section 2 above, the capacitance value depends on the tatic permittivity between the two parallel plates. Static permittivity varies depending on the material occupying the space between the plates. Thus, capacitance value could be used to indicate complete release of accelerometer fingers after HF etch. Partially released fingers would have a substantially higher capacitance value due to the presence of SOG in between the fingers. Test results indicate that partially released encapsulated accelerometers have capacitance values around 10 pF, compared to 1 to 2 pF for completely released devices. For the 11 encapsulated accelerometers tested, capacitance values were between 0.8 to 1.8 pF. The values before and after encapsulation are very close, generally within less than 10% variation (Figure 9). This indicates complete release of the movable elements. The test was repeated on identical samples after 3,000 hours to inspect the condition of the packaged ambient. It is observed that the capacitance values remain unchanged. This indicates the sustainability of the ambient within the package for a prolonged period of time.

## 5. Conclusions

This work has presented a novel wafer level packaging technique for isolatable MEMS devices using a simple and economical CMOS compatible microfabrication processes. The encapsulation uses spin-on glass (SOG) as the sacrificial layer, annealed SU-8 polymer as the base layer, RF sputtered silicon as the main structural layer, eutectic gold-silicon alloy as the seal layer, and high strength liquid crystal polymer (LCP) thermoplastic as the outer encapsulant layer. A robust encapsulation is produced only by using and carefully sequencing these CMOS compatible materials and processes, without the need for expensive packaging equipments. The encapsulation could be fabricated on prereleased as well as released MEMS devices. The completed encapsulation consists of a 25 μm thick silicon encapsulation, 8.75 μm thick base layer, 1 μm thick gold layer, and approximately 210 μm thick LCP layer built on movable elements of MEMSTech 50g accelerometer. The layout was 1,900 μm by 1,900 μm square, with a 1,250 μm by 1,250 μm inner cavity area. The encapsulation has 5 μm clearance between the movable elements and the encapsulation structure. Prior to LCP encapsulant application, the silicon encapsulation is tested for surface uniformity using *Tencor* surface profiler. It is observed that the fabrication process has yielded an encapsulation structure with an average thickness of approximately 40 μm with no detrimental cracks. Subsequently, the encapsulated devices are broken in half to inspect the internal features of the encapsulation. Scanning electron microscope (SEM) inspection indicates that the accelerometer fingers are completely released upon sacrificial SOG removal using hydrofluoric acid (HF). Moreover, comparable capacitance values before and after encapsulation indicate complete release of the movable fingers. Completed encapsulation structure with LCP outer encapsulant coating is tested for strength using *NanoTest* indenter system. Nanoindentation test results indicate that the glob top outer encapsulant is sufficiently strong to endure transfer molding process. The encapsulation technique developed in this work has successfully produced robust MEMS packaging using cheap and simple CMOS compatible processes. Solution for some of the common MEMS packaging problems such as process compatibility, custom and complex packaging equipments required, and high cost has been offered for isolatable MEMS devices.

## Figures and Tables

**Table 1.** Total etch time needed to completely release the movable elements of encapsulated MEMSTech 50g accelerometer in 5% HF solution.

**Section**	**Section length (μm)**	**Etch rate (μm/min)**	**Etch time (min)**
A	675	8.5	79
B	20	8.5	2.3
C	2.5	5.6	0.5
D	20	8.5	2.3
**Total etch time**			**84.1**

**Table 2.** Thickness of layers constituting the encapsulation structure.

**Layer**	**Thickness (μm)**
Air gap	5.0
SU-8	8.75
Sputter silicon	25.0
Sputter gold	1.0
**Total thickness**	**39.75**

**Table 3.** Load-depth correlation and other mechanical properties of the cured RTP 3485-1 LCP outer encapsulant.

**Parameter**	**Sample number**						

	1	2	3	4	5	6	7
Maximum indent depth (nm)	2020.46	2007.89	2018.24	2012.27	2007.63	1998.56	2004.01
Maximum load (mN)	5.69	5.39	5.66	5.69	5.49	5.87	6.02
Plastic depth (nm)	1824.74	1812.85	1815.29	1806.51	1820.11	1799.40	1798.38
Hardness (GPa)	0.70	0.67	0.70	0.71	0.68	0.74	0.76
Reduced modulus (GPa)	32.31	30.53	31.78	31.43	30.57	30.42	31.53
